# Increased Hypothalamic Inflammation Associated with the Susceptibility to Obesity in Rats Exposed to High-Fat Diet

**DOI:** 10.1155/2012/847246

**Published:** 2012-07-11

**Authors:** Xiaoke Wang, Aiguo Ge, Mengjie Cheng, Fangfang Guo, Min Zhao, Xiaoqi Zhou, Liegang Liu, Nianhong Yang

**Affiliations:** ^1^Department of Occupational and Environment Health, School of Public Health, Nantong University, Nantong 226019, China; ^2^Department of Nutrition and Food Hygiene, MOE Key Lab of Environment and Health, School of Public Health, Tongji Medical College, Huazhong University of Science & Technology, 13 Hangkong Road, Wuhan 430030, China

## Abstract

Inflammation has been implicated in the hypothalamic leptin and insulin resistance resulting defective food intake during high fat diet period. To investigate hypothalamic inflammation in dietary induced obesity (DIO) and obesity resistant (DIO-R) rats, we established rat models of DIO and DIO-R by feeding high fat diet for 10 weeks. Then we switched half of DIO and DIO-R rats to chow food and the other half to high fat diet for the following 8 weeks to explore hypothalamic inflammation response to the low fat diet intervention. Body weight, caloric intake, HOMA-IR, as well as the mRNA expression of hypothalamic TLR4, NF-**κ**B, TNF-**α**, IL-1**β**, and IL-6 in DIO/HF rats were significantly increased compared to DIO-R/HF and CF rats, whereas IL-10 mRNA expression was lower in both DIO/HF and DIO-R/HF rats compared with CF rats. Switching to chow food from high fat diet reduced the body weight and improved insulin sensitivity but not affecting the expressions of studied inflammatory genes in DIO rats. Take together, upregulated hypothalamic inflammation may contribute to the overeating and development of obesity susceptibility induced by high fat diet. Switching to chow food had limited role in correcting hypothalamic inflammation in DIO rats during the intervention period.

## 1. Introduction

The prevalence of obesity is growing rapidly and has become a major public health problem worldwide [1]. Associated with the pathogenesis of type 2 diabetes, hyperlipidemia and the increased risk of cardiovascular mortality [2, 3], obesity can only develop when energy intake exceeds energy expenditure. Dietary fat has been shown to influence eating behavior and the development of obesity [4, 5]. However, both human beings and rodents appear to be different in developing obesity when they exposed to high-fat diet, described as dietary induced obesity (DIO) and dietary induced obesity resistant (DIO-R) [6, 7]. The potential mechanism relating the susceptibility to obesity induced by high-fat diet has not been clearly elucidated.

Obesity is considered to be a chronic low-grade inflammatory state [8]. Many studies have revealed that increased inflammatory response in hypothalamus produces insulin and leptin resistance contributing to the defective food intake both in genetic or dietary fat-induced obesity [9–11]. Recently, considerable evidence suggests the inflammatory response to dietary fat is mediated by TLR (Toll-like receptor) signaling, which result in the activation of NF-*κ*B and production of inflammatory cytokines, such as IL-1*β*, IL-6, and TNF-*α* [12–14]. TLRs are pattern-recognition receptors which provide the first line of host defense, and four members of the TLR family including TLR1, 2, 4, and 6, are reported to recognize lipid containing motifs [12, 13]. Emerging study proposed that TLR4 acts as a predominant molecular target for saturated fatty acids in the hypothalamus and triggers the intracellular signaling network that induces an inflammation response and that ultimately results to overeating and obesity [15]. Moreover, genetic deletion designed to disrupt TLR4 signaling protects against high-fat diet-induced obesity [16]. Recent investigations also demonstrated that hypothalamic IKKB/NF-*κ*B was upregulated by high-fat diet and associated with diminished hypothalamic insulin and leptin signal transduction [17, 18]. Further, experimental and genetic interventions that block the hypothalamic NF-*κ*B signaling reversed hypothalamic insulin and leptin resistance and was associated with reduced food intake and weight loss in the high-fat-induced obesity [17]. These data collectively implicated that the activation of hypothalamic inflammation is necessary and sufficient for the control of food intake and likely involved in the mechanism underlying the pathogenesis of obesity susceptibility during high-fat diet feeding.

We have previously shown that upregulated hypothalamic NPY and Y1, Y2, and Y5 receptor gene expressions were closely associated with being predisposed to obesity and overeating of DIO rats [19]. To our knowledge, no studies have been carried out to investigate the involvement of hypothalamic inflammation on the different susceptibility to obesity between DIO and DIO-R rats and their responses to chow food. The main aim of the present study is to compare the expressions of TLR4, NF-*κ*B, and inflammatory cytokine in the hypothalamus between DIO and DIO-R rats for investigating the underlying mechanism related to obesity susceptibility induced by high-fat diet and their respective responses to low-fat dietary intervention.

## 2. Materials and Methods

### 2.1. Animals, Diets, and Experimental Protocols

The experimental protocol was approved by the Animal Care and Use Committee of Huazhong University of Science and Technology. Fifty-five six-week-old outbred male SD rats (purchased from Shanghai Sippr-BK Laboratory Animal Co. Ltd.) weighing 150–160 g were housed individually with regulated temperature (22 ± 5°C) and humidity (50 ± 10%) on a daily cycle of 12 h light and darkness (08:00–20:00 h). All rats were allowed ad libitum access to water and food throughout the experimental period.

After a week acclimation, tail blood was collected and serum was stored under −80°C for further assay. Then the rats were randomly divided into two groups: the HF group (*n* = 45) was placed on a high-fat diet containing 4.62 kcal/g (49.85% fat, 20.00% protein, and 30.15% carbohydrate) and the CF group (*n* = 10) remained on normal laboratory chow food (purchased from Tongji Medical College Laboratory Animal Center, Wuhan, China) containing 3.29 kcal/g (13.68% fat, 21.88% protein, and 64.44% carbohydrate). Dietary intake was recorded daily and body weight was measured weekly in the morning throughout the study. After 10 weeks of free access to their corresponding diet, rats in HF group with body weights more than$\;\;\overset{-}{x}$ + 1.96 s of CF group were designated as DIO and those with body weight less than $\overset{-}{x}$ + 1.0 s of CF group were designated as DIO-R rats. Then one half of the DIO and DIO-R rats were switched to chow food and the other half were kept on HF diets for the following 8 weeks. All rats were provided water and food ad libitum. Terminally, all animals were killed after 12 h fasting between 08:00 and 11:00. Trunk blood was collected and centrifuged and serum was stored for further use. Perirenal and epididymal white adipose tissue was dissected and weighed. Hypothalamus was located and isolated according to brain coronal plane iconography of rat and related articles [20, 21]. Samples of hypothalamic tissues were snap frozen in liquid nitrogen immediately and stored at −80°C for RNA extraction.

### 2.2. Fasting Serum Glucose and Insulin Assay

Serum glucose level was assayed by an enzymatic kit (Nanjing Jiancheng Bioengineering Institute, Nanjing, China) and serum insulin level was measured by radioimmunoassay kits (Beijing Chemclin Biotechnology Corporation Limited, Beijing, China). HOMA-IR was calculated by (fasting serum glucose × fasting serum insulin)/22.5. All the analyses were conducted in duplicate.

### 2.3. RNA Preparation and mRNA Quantification by Real-Time PCR

Total RNA was isolated from hypothalamic tissue using the Trizol Reagent Kit (Invitrogen, USA) following the manufacturer's instruction. The total amount of RNA was measured by spectrophotometry at an absorbance of 260 nm and designated the purity valid if the ratio of A260/A280 was in the range from 1.8 to 2.0. The integrity of the RNA was checked by denaturing agarose gel electrophoresis and ethidium bromide staining. 3.0 *μ*g of the total RNA was reverse transcribed by revert Aid First Strand cDNA synthesis kit (Fermentas, CA, USA). The abundances of TLR4, NF-*κ*B, TNF-*α*, IL-1*β*, IL-6, IL-10, and glyceraldehyde-3-phosphate dehydrogenase (GAPDH) mRNA were analyzed by real-time polymerase chain reaction (PCR) in the 7900 HT real-time PCR system (Applied Biosystems, Foster, CA, USA). Real-time PCR was performed using the SYBR Premix Ex Taq (TaKaRa Bio Inc.) according to the manufacturer's instructions. Reactions were performed in a total volume of 10 *μ*L containing 1 *μ*L cDNA, 0.2 *μ*L ROX reference Dye, 0.2 *μ*M of each primer and 5 *μ*L of the SYBR Green reaction mix. The amplification protocol was as follows: 95°C/10 s (95°C/5 s, 60°C/30 s) × 40. Following amplification, a dissociation curve analysis was performed to insure purity of PCR product. The specific sense and antisense primers were shown as follows. TLR4 (110 bp), sense: 5′-GCA GAA AAT GCC AGG ATG ATG-3′, antisense: 5′-AAG TAC CTC TAT GCA GGG ATT CAA G −3′; NF-*κ*B (167 bp), sense: ATC TGT TTC CCC TCA TCT TTC, antisense: GTG CGT CTT AGT GGT ATC TGT G; TNF-*α* (145 bp): sense: 5′-GGA AAG CAT GAT CCG AGA TG-3′, antisense: 5′-CAG TAG ACA GAA GAG CGT GGT G-3′, IL-1*β* (131 bp): sense: 5′-TGT GAT GTT CCC ATT AGA C-3, antisense: 5′-AAT ACC ACT TGT TGG CTT A-3′; IL-6 (100 bp), sense: 5′ TTG CCT TCT TGG GAC TGA TG 3′, antisense: 5′ ACT GGT CTG TTG TGG GTG GT 3′′; IL-10 (102 bp): sense: 5′-AGG GTT ACT TGG GTT GC-3′, antisense: 5′-ATG CTC CTT GAT TTC TGG-3′; GAPDH (140 bp): sense: 5'-GCA AGT TCA ACG GCA CAG-3′, antisense: 5′-GCC AGT AGA CTC CAC GAC AT-3′. Standard curves for each primer pair were generated by serial dilutions of cDNA from a reference sample and used for regression analyses. All PCR assays were performed in triplicate. The variance of the triplicate measurements was <1%. Results were analyzed using the standard curve method [22] by the SDS (sequence detection systems) software. The data was expressed as the relative levels of mRNA after normalized with GAPDH.

### 2.4. Statistical Analysis

The results were expressed as mean ± SEM. Statistical comparisons were assessed by multivariate analysis (MANOVA), followed by Bonferroni post hoc analysis using the SPSS 13.0 statistical package (SPSS Inc., Chicago, IL, USA). In all analyses, a two-tailed probability of less than 5% (*P* < 0.05) was considered to be statistically significant.

## 3. Results and Discussion

**Figure 1:**
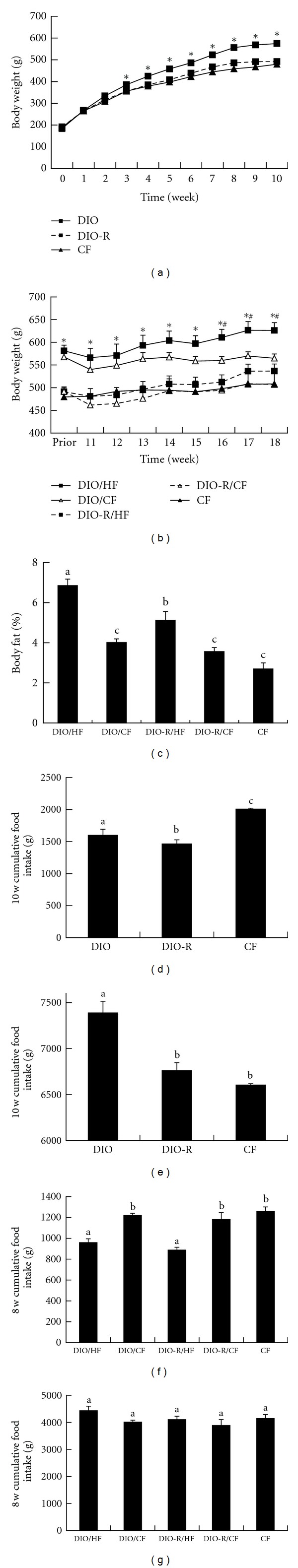
Body weight in DIO, DIO-R, and CF groups during the 10 weeks (a) and the change over 8 weeks dietary intervention (b) was observed weekly. Body fat in each group was measured at 18th (c). Cumulative food intake (d and f), cumulative energy intake (e and g) were obtained in different periods and results expressed as mean ± SEM.  *P < 0.05 for DIO versus DIO-R or CF; ^#^ *P* < 0.05 for DIO/HF versus DIO/CF. Groups sharing different letters above the bars mean statistically significant differences (*P* < 0.05), while those denoted by the same letters are insignificant. DIO: dietary induced obesity; DIO-R: dietary induced obesity resistant; CF: chow food; DIO/HF: DIO on HF; DIO/CF: DIO on CF; DIO-R/HF: DIO-R on HF; DIO-R/CF: DIO-R on CF.

At the end of 10 weeks, rats fed high-fat diet had a wide distribution in body weight and weight gain. 18 of the 45 were designated as DIO and 12 of which were DIO-R and the remains with weight between DIO and DIO-R were excluded from experiments. As presented in Figure 1(a), the body weight in DIO rats was significantly higher than DIO-R or CF rats beginning from 3 weeks to 10 weeks, whereas no significant difference was observed between DIO-R and CF rats throughout the experiment period. During the following 8 weeks, changing the diet to standard chow from high-fat diet reduced the weight gain in DIO rats but not in DIO-R rats (Figure 1(b)). At the end of the experiment, the percentage of fat mass in DIO/HF rats was significantly higher than that in DIO-R/HF and CF rats, while DIO/CF and DIO-R/CF had a lower percentage of fat mass compared with their counterparts on high-fat diet, no significant difference was found between DIO/CF, DIO-R/CF, and CF rats (Figure 1(c)).

Cumulative food intake and energy intake during the first 10 weeks and the following 8 weeks were compared among groups. Over the first 10 weeks, DIO rats had greater food intake than DIO-R rats though they all had free access to high-fat diet (Figure 1(d)). When food intake was calculated as energy intake for the different energy density between chow food and high-fat diet, DIO rats had greater energy intake than DIO-R and CF rats, whereas no significant difference was found between DIO-R and CF groups (Figure 1(e)). After shifting to chow food from high-fat diet, the food intake in both DIO/CF and DIO-R/CF were increased compared with their respective counterpart continued on high-fat diet (Figure 1(f)). No significant difference in energy intake was found among groups during the intervention period (Figure 1(g)).

At the initiation of this experiment, there were no differences among groups in serum glucose, insulin, and HOMA-IR. The serum glucose, insulin, and HOMA-IR in DIO rats were higher than DIO-R and CF rats, no difference was found between DIO-R and CF rats after 10 weeks. Switching to standard chow from the high-fat diet, serum insulin and HOMA-IR in DIO/CF rats were finally reduced compared with DIO/HF rats and similar to that in CF rats, while no difference was found between DIO-R/CF and DIO-R/HF rats (Table 1).

**Figure 2:**
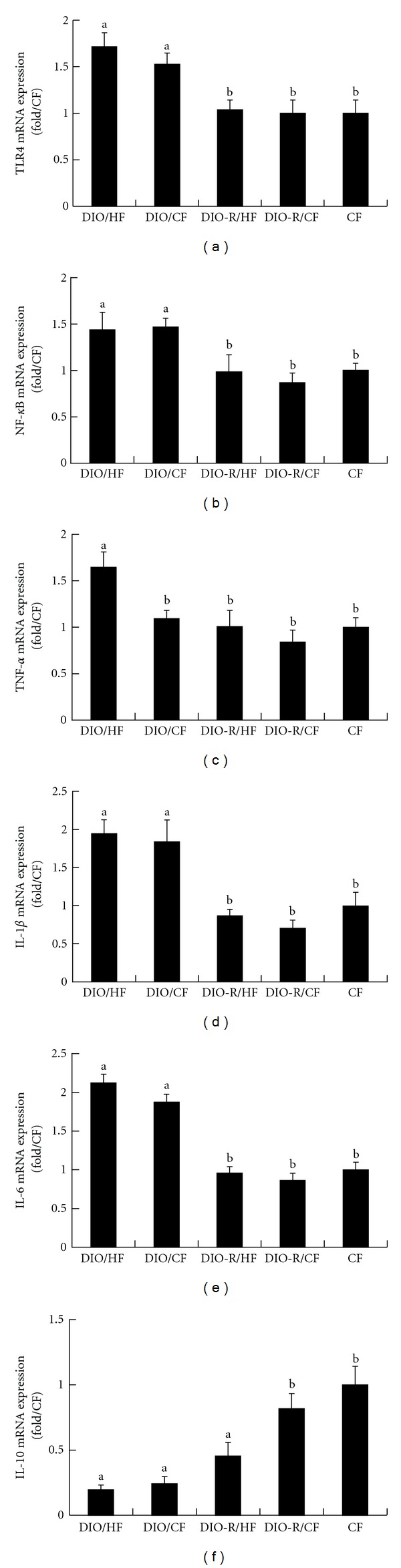
Relative mRNA expression of TLR4 (a), NF-*κ*B (b), TNF-*α* (c), IL-1*β* (d), IL-6 (e), and IL-10 (f) in hypothalamus was measured by real-time PCR in each group at 18th week. All results were normalized to GAPDH and expressed as arbitrary units. Data was shown as mean ± SEM. Groups sharing different letters above the bars mean statistically significant differences (*P* < 0.05), while those denoted by the same letters are insignificant. DIO: dietary induced obesity; DIO-R: dietary induced obesity resistant; CF: chow food; DIO/HF: DIO on HF; DIO/CF: DIO on CF; DIO-R/HF: DIO-R on HF; DIO-R/CF: DIO-R on CF.

As shown in Figures 2(a) and 2(b), the hypothalamic expression of genes encoding TLR4 and NF-*κ*B mRNA were significantly increased in DIO/HF rats as compared to DIO-R/HF and CF rats, no significant difference was detected between DIO-R/HF and CF rats. Changing the diet from the high-fat diet to chow food did not affect the level of hypothalamic TLR4 and NF-*κ*B mRNA expression both in DIO and DIO-R rats. Hypothalamic TNF-*α*, IL-1*β*, and IL-6 mRNA expressions in DIO/HF rats were higher compared with the DIO-R/HF and CF rats, no difference was found between DIO-R/HF and CF rats. Switching diet to chow food from high-fat diet did not affect the TNF-*α*, IL-1*β*, and IL-6 expression in hypothalamus of DIO rats (Figures 2(c), 2(d) and 2(e)). The anti-inflammatory cytokine IL-10 mRNA expression was lower both in DIO/HF and DIO-R/HF rats compared with CF rats, but only restored in DIO-R/HF rats by switching to chow food (Figure 2(f)).

This study compared hypothalamic inflammation between high-fat diet-induced obese and obese-resistant rats and their responses to chow food intervention. The main finding is as follows: (1) DIO/HF rats but not DIO-R/HF rats exhibited significant increase in TLR4, NF-*κ*B, TNF-*α*, IL-1*β*, and IL-6 mRNA expression as well as energy intake when compared to CF controls. (2) Switching from high-fat diet to chow food failed to affect hypothalamic TLR4, NF-*κ*B, TNF-*α*, IL-1*β*, and IL-6 mRNA expression both in DIO and DIO-R rats except an increased IL-10 mRNA expression in DIO-R rats. (3) DIO/CF rats remained higher hypothalamic TLR4, NF-*κ*B, TNF-*α*, IL-1*β*, and IL-6 mRNA expression than DIO-R/CF rats.

It is generally accepted that obesity results from the complex interaction of genetic components that predispose to obesity and environment which facilitates the development of obese phenotype [23]. Although high-fat diet is among the most important environment factors leading to obesity, both rodent and human beings showed different susceptibility to obesity in response to a high-fat diet [6, 24] and models of diet-induced obesity are commonly used to study the human obesity in the context of an environment where energy-dense foods and diets are highly available. Our present study confirmed that the outbred SD rats exhibited different phenotype after exposed to high-fat diet. The increased energy intake appeared to be primarily responsible for the increased weight gain in DIO rats on high-fat diet ad libitum, while DIO-R rats exposed to high-fat diet compensated for the increased energy density of the high-fat diet by eating significantly less, similar to the previous studies [25, 26].

There is an intimate relationship between the immune and metabolic systems during the evolutionary period and might allow nutrients to act through pathogen-sensing pathway for storing energy and fighting off the infection [27]. In the presence of abundance nutritional surplus, this once advantageous immune-metabolic system contributes to the excess energy intake and adiposity. TLRs, especially TLR4, were shown to act as the receptor both for pathogens and saturated fatty acid [28, 29], giving rise to interaction between immune response and metabolic system. Recently, investigators proposed that activated hypothalamic TLR4/NF-*κ*B and the production of inflammatory cytokines were implicated in the defective food intake after exposure to dietary fat [9, 15, 17]. In the present study, we found that hypothalamic TLR4, NF-*κ*B, TNF-*α*, IL-1*β*, and IL-6 mRNA expressions were significantly higher in DIO/HF rats compared with DIO-R/HF and CF rats. Similar to the results in our study, Zhang et al. [17] have shown that the hypothalamic NF-*κ*B activity was 5- to 6-fold higher in ob^+/+^ mice (a hyperphagic obesity) compared with the wild type controls even both fed normal chow, implicating that higher inflammation response in hypothalamus was associated with obesity susceptibility. An observation from a pair-feeding model showed that the rats fed the high-fat diet had higher IKKB expression and reduced I*κ*B*α* expression in hypothalamus compared with the rats that consumed the same amount of calories from the low-fat diet [18], which conflicts with the result in our study that there is no significant difference in hypothalamic inflammation between DIO-R/HF rats and CF rats even though they consumed the same amount of calories. One potential explanation for this phenomenon is the use of a different rat model and DIO-R/HF rats may have the inherent physicochemical properties to normal the central inflammatory response for governing energy balance. Combined with insulin resistance and increased energy intake in DIO/HF rats, these findings suggested that upregulated hypothalamic inflammation may lead DIO/HF rats to consume more energy and obtains more weight than DIO-R/HF rats when fed with the same high-fat diet ad libitum, while DIO-R/HF rats could appropriately adjust caloric intake by preventing increased hypothalamic inflammation. And the reason why DIO-R/HF rats could automatically prevent the increased hypothalamic inflammatory response in DIO-R/HF rats needs further study.

Low-fat diet has been investigated extensively for reversibility of chronic high-energy diet-induced obesity [30, 31]. However, the result was controversial for the genetic background, duration of high-fat diet and food palatability [32]. In the present study, DIO rats switched to chow (DIO/CF), lost their body weight, and finally improved insulin sensitivity, concurring with the previous study [33–35], whereas there was no significant alteration in either hypothalamic inflammatory or anti-inflammatory cytokine between the DIO/HF and DIO/CF groups. Combined with no difference in energy intake between the DIO/HF and DIO/CF groups during the intervention period, this result implicates that the weight loss and improved insulin sensitivity in DIO/CF rats may be mediated mainly through reduced fat content in chow food, independent of the total energy intake by correcting hypothalamic inflammation. Previous studies have revealed that hypothalamic leptin resistance was associated with inflammation [15, 36] and reversed by 20 weeks of low-fat diet feeding [33], suggesting that the hypothalamic inflammation could be improved by low-fat diet. However, the studied hypothalamic inflammation genes were not altered after chow food intervention in the present study. One potential explanation is that 8 weeks intervention period in our study is not enough to ameliorate the hypothalamic inflammation and additional research of prolonged intervention is warranted.

It is believed that inflammation produced by hypertrophied adipose tissues is a strong driving force for the development of type 2 diabetes [37, 38]. Recent studies demonstrated that hypothalamic inflammation induced by chronic high-fat diet could not only cause feeding and body weight changes, but also employ body weight-independent manners to cause systemic glucose intolerance [17, 39]. The mechanism underlying is possibly related to induction of central leptin and insulin resistance. Further studies are needed to elucidate the role of hypothalamic inflammation in the induction of central leptin and insulin resistance.

## 4. Conclusions

Taken together, this study showed that the DIO rats appear to have higher energy intake and greater fat storage and body weight than the DIO-R rats when both have free access to high-energy-density diet. Excessive energy intake and body weight gain in DIO rats was attributed to the activated hypothalamic inflammation induced by high-fat diet. Switching to low-fat chow failed to recover hypothalamic inflammation, although body weight and fat pad were decreased and insulin sensitivity was improved in DIO/CF rats. The limitation of this study is that we assessed the contribution of inflammation response to feeding behavior and obesity phenotype after DIO and DIO-R had already occurred. The inflammatory response evolved over time after exposure to high-fat diet and early response in hypothalamus which may trigger distinct intermediate responses that in turn lead to different late responses. Further studies with different feeding periods and regimes are warranted.

## Figures and Tables

**Table 1:** Fasting serum level of glucose, insulin, and HOMA-IR.

	DIO/HF	DIO/CF	DIO-R/HF	DIO-R/CF	CF
FPG (mmol/L)					
0 W	2.61 ± 0.14	2.93 ± 0.10	2.94 ± 0.29	2.86 ± 0.14	2.94 ± 0.04
10 W	5.16 ± 0.12^ab^	5.27 ± 0.11^ab^	4.24 ± 0.09	4.10 ± 0.13	4.12 ± 0.07
18 W	4.96 ± 0.10	4.73 ± 0.20	4.62 ± 0.21	4.60 ± 0.09	4.84 ± 0.10
FINS (*μ*IU/mL)					
0 W	27.02 ± 1.01	23.50 ± 1.29	25.12 ± 1.18	23.70 ± 1.62	23.01 ± 1.13
10 W	54.59 ± 1.39^ab^	55.31 ± 1.06^ab^	43.47 ± 1.31^a^	45.98 ± 1.36^a^	30.18 ± 1.18
18 W	60.16 ± 2.52^abc^	43.55 ± 2.69	45.87 ± 1.01	41.17 ± 2.12	31.66 ± 1.83
HOMA-IR					
0 W	3.24 ± 0.51	3.16 ± 0.20	3.18 ± 0.42	3.11 ± 0.39	3.14 ± 0.03
10 W	12.56 ± 0.85^ab^	12.64 ± 1.12^ab^	8.32 ± 0.08	8.37 ± 1.02	6.52 ± 1.96
18 W	13.36 ± 1.06^abc^	9.25 ± 1.42	9.31 ± 1.03	8.52 ± 1.19	6.91 ± 0.45

Data was shown as mean ± SEM. ^a^ *P* < 0.05 compared with the CF group; ^b^ *P* < 0.05 compared with the DIO-R/HF group; ^c^ *P* < 0.05 compared with their perspective CF intervention group. FPG: fasting serum glucose; FINS: fasting serum insulin; DIO: dietary induced obesity; DIO-R: dietary induced obesity resistant; CF: chow food; DIO/HF: DIO on HF; DIO/CF: DIO on CF; DIO-R/HF: DIO-R on HF; DIO-R/CF: DIO-R on CF.

## Abbreviations

DIO: Dietary induced obesity
DIO-R: Dietary induced obesity resistant
CF: Chow food
DIO/HF: DIO on HF
DIO/CF: DIO on CF
DIO-R/HF: DIO-R on HF
DIO-R/CF: DIO-R on CF
TLR4: Toll-like receptor-4
NF-*κ*B: Nuclear factor *κ*B
TNF-*α*: Tumor necrosis factor-*α*
IL-1*β*: Interleukin-1*β*
IL-6: Interleukin-6
IL-10: Interleukin-10
Real-time PCR: Real-time polymerase chain reaction.
